# Titanium dioxide nanoparticles elicited agro-morphological and physicochemical modifications in wheat plants to control *Bipolaris sorokiniana*

**DOI:** 10.1371/journal.pone.0246880

**Published:** 2021-02-11

**Authors:** Seema Hassan Satti, Naveed Iqbal Raja, Bilal Javed, Abida Akram, Zia-ur-Rehman Mashwani, Muhammad Sheeraz Ahmad, Muhammad Ikram

**Affiliations:** 1 Department of Botany, PMAS Arid Agriculture University, Rawalpindi, Punjab, Pakistan; 2 University Institute of Biochemistry and Biotechnology, PMAS Arid Agriculture University, Rawalpindi, Punjab, Pakistan; VIT University, INDIA

## Abstract

The current study involves the biogenesis of titanium dioxide nanoparticles (TiO_2_ NPs) by using *Moringa oleifera* Lam. aqueous leaf extract for the reduction of titanium dioxide salt into TiO_2_ nanoparticles. The biosynthesized TiO_2_ nanoparticles were observed by using the UV-visible spectrophotometry, SEM, EDX and XRD analytical methods. It was confirmed that the nanoparticles are crystalline and exist in the size range of 10–100 nm. The FTIR analysis confirmed the presence of O-H (hydrogen bonding), N-H (amide), C-C (alkanes) and C-I (Iodo-stretch) functional groups responsible for the stabilization of nanoparticles. Various concentrations (20, 40, 60 and 80 mg/L) of TiO_2_ NPs were applied exogenously on wheat plants infected with a fungus *Bipolaris sorokiniana* responsible to cause spot blotch disease at different time intervals. The measurement of disease incidence and percent disease index showed the time-dependent response and 40 mg/L was reported a stable concentration of TiO_2_ NPs to reduce the disease severity. The effects of biosynthesized TiO_2_ NPs were also evaluated for agro-morphological (leaf and root surface area, plant fresh and dry weight and yield parameters), physiological (relative water content, membrane stability index and chlorophyll content) and non-enzymatic metabolites (soluble sugar, protein, soluble phenol and flavonoid content) in wheat plants under biotic stress and 40 mg/L concentration of TiO_2_ NPs was found to be effective to elicit modifications to reduce biotic stress. The current study highlights the significant role of biosynthesized TiO_2_ NPs in controlling fungal diseases of wheat plants and thus ultimately improving the quality and yield of wheat plants.

## 1. Introduction

Wheat is considered as a staple food after rice due to its high nutritional value and diverse uses [1, 2]. Despite being the predominant crop in Pakistan, it shares 8.9% of the added value in agriculture and accounts for only 1.6% of GDP [3]. Pakistan still lags in meeting the international average of the wheat yield [4]. Various biotic factors such as fungal diseases result in declining wheat production and result in ~12.4% of yield loss annually all over the world [1, 5]. Among these pathogens, fungi are the main disease-causing agents in wheat. Fungi are not only affecting the yield but also affects the quality of grains and hygiene. The fungal produced mycotoxins affect the total nutritive value of the wheat grains [5]. In Pakistan, different fungal diseases include rusts, blights or spots and smuts, *etc*. are the main reasons for the low production of wheat [6]. A very important and disastrous fungal pathogen of wheat is *Bipolaris sorokiniana* (Sacc.) Shoem and is responsible for almost 20% of yield loss by causing a spot producing disease called spot blotch [6, 7]. The spot blotch disease appears on the lower surface area of the leaves with discrete, elongate and black-brown lesions [1]. It can cause infection on the entire wheat plant in the form of the blight of seedlings, rotting of roots, lesions of spot blotch and black or brown points on the grain, subsequently resulting in huge damage in the quality and the quantity of the crop. It was earlier reported that the spot blotch was the most prevalent disease of important commercial varieties cultivated in the fields and none of the variety was found resistant to the disease [6]. The use of chemical methods to control pathogen proliferation and crop management has hazardous effects on the crop and ultimately affect human health. The use of modern technologies can be exploited to limit harmful agrochemicals [8]. Nanotechnology can play a vital role in dealing with these problems [9–11]. Recently, titanium dioxide (TiO_2_) has extensively been used as an environment friendly and clean photocatalyst due to its optical characteristics, chemical stability and non-toxic nature [12–14]. The titanium dioxide nanoparticles have also shown significant antimicrobial and antibacterial activity [15, 16], which is of high importance due to the developing resistance of microbes against antibiotics and the development of the resistance varieties [17].

It is considered that the imbalance in plant synthesis of antioxidants and quenching system may cause oxidative damage that results in the production of reactive oxygen species (ROS) and ultimately affect the physiological functioning and eventually leads to cell death and decline in the crop yield [18–20]. For protecting the plant from the dangerous effects of the ROS, the defense system against oxidative damage is activated by enzymatic (superoxide dismutase, peroxidase and catalase) and non-enzymatic antioxidants such as phenolic compounds, proline and flavonoids [21]. Metallic nanoparticles especially TiO_2_ NPs have been reported to activate the antioxidant defense system of the plants [19].

Bio-fabrication of nanoparticles by using the reducing abilities of the plant secondary metabolites has advantages to generate functional nanoparticles to treat microbial resistance [17, 22]. It has advantages to provide ecofriendly synthesis procedure, inexpensive reactants and the fabrication of biocompatible nanomaterials [8, 9, 23]. Plant-based TiO_2_ NPs play a promising role to rapidly absorb on the surface of the pathogen and plant material where they function to kill pathogen in addition to the organogenesis and the growth-promoting response on plants [24, 25].

Only a few scientific studies have reported the role of TiO_2_ NPs to ameliorate the intensity of the disease, their effects in altering biochemistry in response to fungal stress and productivity attributes in wheat plants. It was hypothesized that the exogenous applications of TiO_2_ NPs function to control the proliferation of *Bipolaris sorokiniana* fungus by improving the tolerance of wheat plants through modulating physiological and biomolecular pathways. This study aimed to evaluate the *in situ* antifungal effects of the biosynthesized TiO_2_ NPs in response to *Bipolaris sorokiniana* (Spot blotch) stress, and evaluation of the morphological and physicochemical parameters to assess the development of the resistance in the wheat plants.

## 2. Materials and methods

### 2.1. Preparation of plant leaf extract

*Moringa oleifera* leaf aqueous extract was prepared to synthesize TiO_2_ NPs. The plant was collected from Islamabad Margalla Hills (33.747774, 73.007782). The plant fresh leaves were soaked in freshwater and then washed thoroughly with the distilled water to remove dust particles. The leaves were subjected to air drying and grounded to form the fine powder. The 25 mg of the prepared powder was dissolved in 100 mL of Milli-Q^®^ water and stirred for almost 40 minutes with a magnetic stirrer. The final solution was filtered and used fresh [23].

### 2.2 Synthesis of TiO_2_ NPs

About 20 ml of the plant aqueous extract was mixed with 1 M of TiO_2_ salt solution (TiO(OH)_2_, Sigma-Aldrich) and subjected to stirring for 24 hours at room temperature for the synthesis of TiO_2_ NPs. The change in the color of the reaction mixture from white to pink-brown indicated the production of TiO_2_ NPs. The reaction mixture was centrifuged at 15,000 rpm for 15 minutes for the separation of TiO_2_ NPs. The resultant pellet was dried via SpeedVac concentrator and stored for further studies [12].

### 2.3. Optical and morphological characterization of nanoparticles

#### 2.3.1. UV-visible spectrophotometry

The synthesis of TiO_2_ NPs was confirmed by using the UV-Visible spectrophotometer and the absorbance was recorded in the range of 200 to 900 nm of the light wavelength [22].

#### 2.3.2. Scanning Electron Microscopic analysis (SEM)

The SEM technique was used to morphologically analyze the structure of biosynthesized TiO_2_ NPs. The SEM SIGMA was operated at 5 kV and magnification was set at ×10 k. A drop coating method was used to prepare the sample on a carbon-coated copper grid. This sample was dried under a mercury lamp for 5 minutes and the excessive solution was removed by using the blotting paper. The surface images of nanoparticles were collected at various magnifications [26].

#### 2.3.3. Energy-dispersive X-ray spectroscopy analysis (EDX)

The elemental analysis of the bio-synthesized TiO_2_ NPs was done by the EDX instrument. The sample was prepared by placing the synthesized NPs on thin carbon films [17].

#### 2.3.4. X-ray diffraction spectroscopic analysis (XRD)

The crystalline or amorphous nature and the structure of the TiO_2_ NPs were studied by using the XRD technique. The powdered sample was placed on a Shimadzu XRD-6000 and set in the range of 5°-50° at a 2θ angle. Debye-Scherrer’s equation (D = Kλ / βcos θ) was applied to determine the average size of the NPs. Here, the K indicates the shape factor or Scherrer constant, λ denotes the X-ray wavelength, and β indicates the full width in radius at half maximum, and θ is the Bragg’s angle [8].

#### 2.3.5 Fourier Transform Infrared Spectroscopy (FTIR)

The FTIR analysis was performed to study the functional groups that are responsible for the formation of TiO_2_ NPs synthesized by *Moringa oliefera* leaf aqueous extract. The dried TiO_2_ NPs powder was pelleted with the potassium bromide and FTIR spectrum was obtained using Perkin-Elmer FTIR-Spectrum with wavenumber in the range of 400 cm^-1^ to 4000 cm^-1^.

### 2.4. Preparation of the fungal inoculum

*B*. *sorokiniana* was collected from the wheat varieties infected with the mother culture of the most virulent strains of *B*. *sorokiniana*. The Potato dextrose agar (PDA) medium was used to maintain the growth of the pathogen till further use. The inoculated agar plates were placed in an incubator at 23°C for 10 days until the maximum growth of the fungus was obtained. *B*. *sorkiniana* inoculum was prepared in suspension by taking 12 days old culture and scraping its surface and finally dissolved in sterilized water. This prepared suspension was placed on the shaker incubator at room temperature to obtain the pure conidial culture. The excess of the agar medium was removed by filtering the suspension through a cheesecloth. The concentration of conidia was adjusted to 8000 conidia per mL with the help of a Hemocytometer. The clustering of conidia was prevented by using Tween-20 [6, 16].

### 2.5. Preparation of glasshouse experiments and treatment plan

To determine the antifungal activity of biosynthesized TiO_2_ NPs on wheat plants against *Bipolaris sorokiniana* a glasshouse experiment was conducted. Several earthen pots having 10 kg of capacity were filled with the sterilized soil. The sandy loam soil was used to conduct experiments. The composition of soil by weight was about sand (40%), silt (20%) and clay (40%). A fungus susceptible wheat variety Galaxy-2013 was obtained from the National Agriculture Research Center, Islamabad. The seeds were treated with 0.1% of mercuric chloride to sterilizing the surface [6]. Five seedlings per pot were kept for further experimentation. The experiments were performed in a completely randomized design (CRD) with each treatment taken in replicates. Initially, the experiments were performed on low concentrations of TiO_2_ NPs to check the effectiveness of the nanoparticles. Then the referenced nanoparticles concentrations were designed for exogenous applications to study their effects to control the growth of fungus versus control plants in greenhouse experiments. The detailed treatment layout or plan is given in Table 1.

**Table 1:** Treatment layout for the assessment of the disease severity and the foliar applications of TiO_2_ NPs in greenhouse experiments.

Treatment	Condition
T0	Control (Healthy wheat plants)
T1	Only Pathogen (*Bipolaris sorokiniana*)
T2	20 mg/L of TiO_2_ NPs + Pathogen
T3	40 mg/L of TiO_2_ NPs + Pathogen
T4	60 mg/L of TiO_2_ NPs + Pathogen
T5	80 mg/L of TiO_2_ NPs + Pathogen

### 2.6. Inoculation of wheat plants with *Bipolaris sorokiniana*

The suspension containing *Bipolaris sorokiniana* spores was used to inoculate the wheat plants by direct spraying the suspension using an atomizer on the leaves of the wheat plants at the booting stage (seven to eight leaves stage at which the flag leaf emerges). A volume of 50 mL of suspension was sprayed as a fine mist on each plant using an atomizer pressurized by an air pump (30 k Pa). The moisture or humidity level was maintained in the greenhouse between 80–90% at the time of inoculation of spores through a mister maintaining the temperature at 18°C. After spraying the spore suspension, the plants were covered by using the transparent bags of polythene and sprayed extensively with autoclaved water for successful pathogen propagation. The temperature was maintained at 16–18°C. The first data was recorded on the 5^th^ day of the inoculation and then after every 10^th^ day (day 15, day 20, day 25 and day 30).

### 2.7. Collection of samples to assess disease severity

The leaf tissues were harvested from the randomly selected wheat plants of three replicates from each treatment. The symptoms indicating the severity of spot blotch caused by *B*. *sorokiniana* was assessed by using a rating scale on a visual basis (Tables 1 and 2). Disease severity was measured on a standard scale of 0–5 for disease as suggested by the reference [6] for leaf spot blotch disease.

**Table 2:** A rating scale for the leaf spot blotch disease.

Number	Level of Symptoms	Resistant Level
0	No symptoms	Resistant
1	1–5% of spots on the leaves	Moderately Resistant
2	6–20% of spots on the leave	Moderately Resistant
3	21–40% of spots on the leaves	Moderately susceptible
4	41–60% of spots on the leaves	Moderately susceptible
5	More than 61% of spots on the leaves	Susceptible

### 2.8. Assessment of disease incidence

Incidence of the disease was determined by the use of the formula given by the reference [6];

$$Disease\;Incidence\;(\%)=\frac{Number\;of\;infected\;plants}{Total\;number\;of\;plants}\times 100$$

The percent disease severity also called disease index was determined by using the formula [6];

$$Percent\;Disease\;Index\;(PDI)=\frac{Disease\;index}{Total\;infected\;plants}\times 100$$

(Spot in scale 1) + (Spot in scale 2) + (Spot in scale 3) + (Spot in scale 4) + (Spot in scale 5)

### 2.9. Measurement of the growth parameters

The shoot length and root length were measured after uprooting the whole plant at the final harvest stage which occurred when the grain reached a suitable moisture level (80–90%). The other yield parameters were also taken at that time.

#### 2.9.1. Measurement of the roots surface area

The roots were separated from the plants of each treatment and surface area was measured using the area meter.

#### 2.9.2. Measurement of the leaf surface area

The leaves were taken at the flag leaf stage to measure the leaf surface area using a leaf area meter (CID, CI-202).

#### 2.9.3 Measurement of the fresh weight and dry weight

The fresh weight of shoots and roots were measured for each treatment and the same plants were dried in a hot air oven for one week at 65°C to record the dry weight [27].

#### 2.9.4 Determination of the yield attributes

Wheat plants were harvested and data was recorded for assessing yield attributes including 100 grains weight (gram), grains per spike and spikes per plant. The sample plants were collected at the final harvest stage as mentioned above.

### 2.10. Measurement of the physiological parameters

The flag leaves were harvested after the heading stage when the head fully emerged from the stem for biochemical analysis of wheat plants treated with various concentrations of TiO_2_ NPs.

#### 2.10.1. Measurement of the relative water content (RWC)

The flag leaves were taken and their fresh weight was measured individually. They were subsequently dipped in distilled water for about 24 hours. After 24 hours, the leaves became turgid and their turgid weight was measured individually. The leaves were then placed in an oven at 65°C for 72 hours to dry them completely and then their dry weights were determined [28].

$$Relative\;Water\;Content=\frac{\left(Fresh\;weight-Dry\;weight\right)}{\left(Saturated\;weight-Dry\;weight\right)}\times 100$$

#### 2.10.2. Determination of the membrane stability index (MSI)

The leaf membrane stability index was determined by using the method described by the reference [29]. The small discs of leaves of about 100 mg were prepared, washed and subjected to heating at 40°C for 30 minutes in a water bath containing 10 mL of double-distilled water. The discs were used to measure the electrical conductivity (CI) by using the electrical conductivity meter. The samples disc were again placed in the boiling water at 100°C without replacing them for 10 minutes and the electrical conductivity (C2) was recorded again. The membrane stability index was computed as follows;

Membrane stability index (MSI) = [1-(C1/C2)] × 100

#### 2.10.3. Determination of the chlorophyll content

The chlorophyll *a*, chlorophyll *b* and the total chlorophyll contents were determined by using the protocol established by the reference [30, 31]. The leaf samples were harvested from the plants for each treatment and subjected to grinding using 10 mL of acetone and then filtered. The resultant filtrate was poured into separate test tubes for every treatment. The absorbance of each treatment sample was recorded at 645 nm, 663 nm and 672 nm wavelength by using a UV-visible spectrophotometer.

Chlorophyll *a* (μg/mL) = 12.7 (A_663_) - 2.7 (A_645_)

Chlorophyll *b* (μg/mL) = 22.9 (D_645_) - 4.7 (D_663_)

Total chlorophyll (μg/mL) = (D_645_ × 1000/34.5)

### 2.11. Measurement of the biochemical parameters

#### 2.11.1. Determination of the soluble sugar

To determine the content of the soluble sugar in each sample, the protocol of the reference [32] was used with slight modifications. According to the method, 0.5 gm of the fresh leaves from each treated plant was put into a test tube and added with 10 mL of 80% ethanol. The leaves were then subjected to heating for an hour followed by adding 18% of phenol in 0.5 mL of the resultant extract. The extract was kept undisturbed for 1 hour at room temperature. The 2.5 mL of H_2_SO_4_ was added to the reaction mixtures and subjected to vigorous stirring followed by recording the absorbance at 490 nm.

$$Sugar\;(\mu g/ml)=\frac{Absorbance\;of\;sample\times Dilution\;factor\times K\;value}{Weight\;of\;fresh\;tissue\;in\;grams}$$

#### 2.11.2. Estimation of total proteins

The total protein content of the leaves was calculated by following the method of reference [33] with certain modifications. The fresh leaf material of around 0.1 gm was subjected to grinding in a mortar and pestle after adding 1 mL of phosphate buffer (pH 7). The resultant homogeneous solution was centrifuged at 30,000 rpm for 15 minutes at room temperature. The supernatant was collected and poured into test tubes. The distilled water was added to the test tubes until the total volume reached 1 mL. The mixture was again subjected to shaking for 10 mins after adding alkaline copper sulfate reagent (1 mL) and 0.1 mL of Folin’s reagent. The whole mixture was placed in an incubator for half an hour. The absorbance of each sample was recorded at 650 nm against 0.1 mL of 0.5 M NaOH blank. Bovine serum albumin (BSA) was used as a standard. The concentration of total soluble proteins was determined with the reference curve using BSA.

#### 2.11.3. Estimation of total soluble phenol content

The total phenolic content of the wheat leaves was assessed from the leaves extract by following the protocol described by the reference [34] with some modifications. Almost 0.7 gm of fresh leaves were taken and 10 mL of methanol was used to homogenize the leaves and then left overnight. The leaves were filtered and the filtrate was diluted with 100 mL of water to serve as a stock solution. The prepared extract was further used to analyze the phenolic content by following the protocol reported by the reference [34] with modifications. According to the method, around 200 μl of the prepared stock solution was poured into a test tube with 1.4 mL of distilled water and 0.1 mL of 50% Folin-Ciocalteu phenol reagent. The sample was left for three minutes and then 23% (w/v) of sodium carbonate (0.4%) was added. The resultant mixture was kept for 2 hours and then subjected to a gentle vortex. The absorbance was recorded at 765 nm. The gallic acid was used as a standard against which the total phenolic content was measured.

#### 2.11.4. Determination of total flavonoid content

The flavonoid content of the leaves was measured by following the method described by the reference [35]. Around 550 mg of leaves were taken and cut into very small pieces. Almost 80% of methanol was added to the leaves to make a fine paste and to achieve a volume of up to 8 mL. This mixture turned into a suspension and was subjected to centrifugation at 20,000× g for 10 minutes. The supernatant was collected and poured into a volumetric flask and around 5 mL of distilled water was added followed by the addition of 0.7 mL of 5% sodium nitrate and 0.6 mL of 10% aluminum chloride. The resultant solution was put to rest for 5 minutes. The solution was again left for 1 minute after adding 3 mL of 1 M sodium hydroxide and 2.5 mL of distilled water and mixed thoroughly. The absorbance was recorded at 510 nm.

#### 2.12 Data analysis

The experiments were performed in triplicate. The significant difference between the control and the treated groups was determined by using Duncan’s multiple range test (p <0.05). The calculations were performed through SPSS^®^ 18.0.

## 3. Results and discussion

### 3.1 Morphological and optical characterization of TiO_2_ NPs

The bio-fabrication of nanoparticles has advantages over the routine physical and chemical methods of synthesis. The phyto-reduction of metal salt results in the synthesis of biologically active nanomaterials that have many biological and medical applications [9].

**Fig 1:**
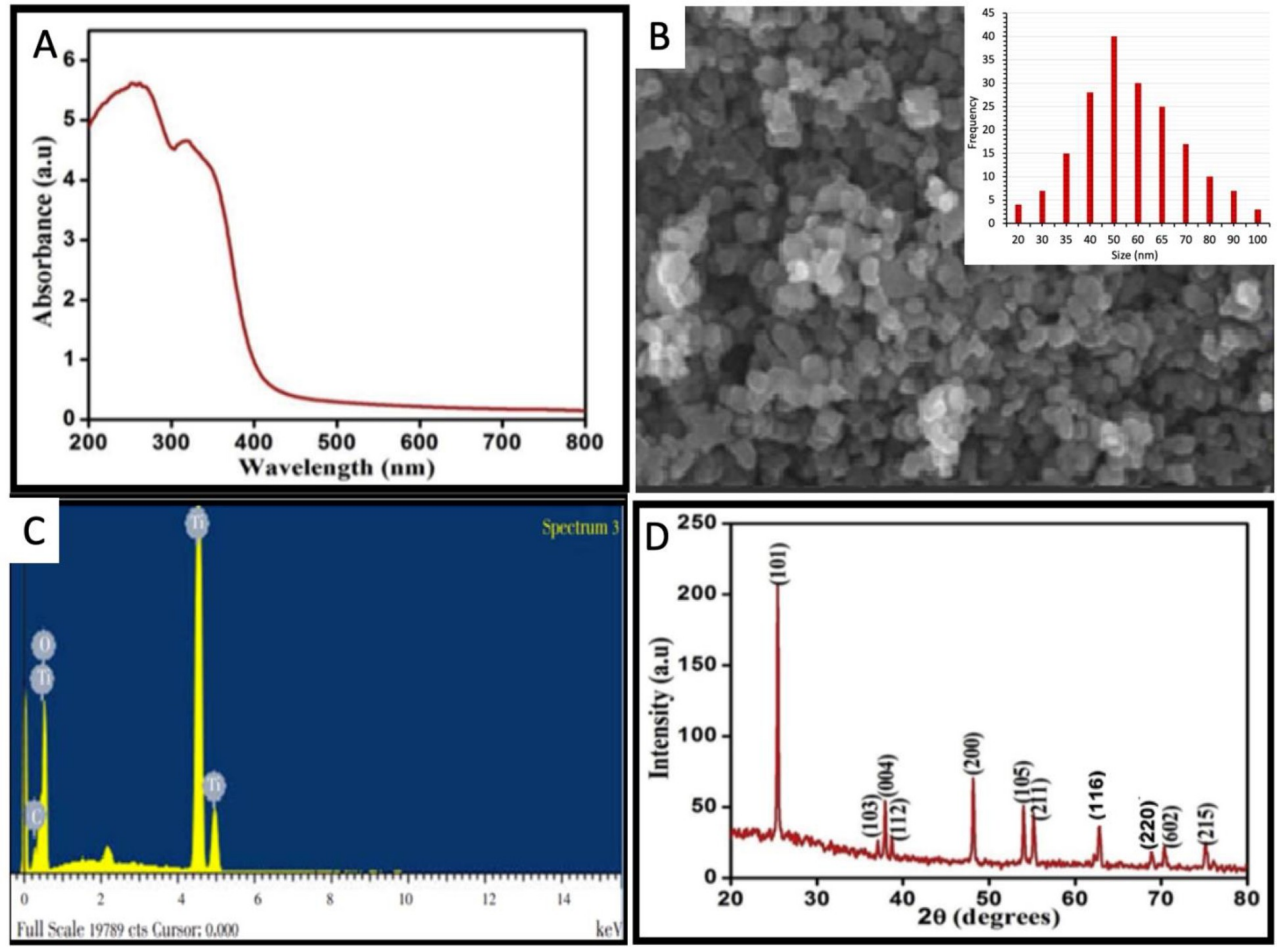
Morphological and optical characterization of bio-fabricated TiO_2_ NPs (a) UV-visible spectrum (b) SEM image; Inset is showing the particle size distribution histogram (c) Elemental composition analysis (d) XRD analysis.

The ongoing study reports the phyto-synthesis of TiO_2_ NPs by using the reducing and stabilizing potential of *M*. *oleifera* leaf aqueous extract. The change in the color of the reaction mixture from milky white to pink-brown was considered as an initial sign to report synthesis. The synthesis of TiO_2_ NPs was confirmed by measuring the absorbance. A characteristic surface plasmon resonance band (SPR) was observed in the range of 240–280 nm to confirm the synthesis of TiO_2_ NPs (Fig 1A). The SPR band is the response of the interaction of the electromagnetic light waves with the oscillating electrons in an electric field [8]. The plasmonic materials are metals or metal-like materials that exhibit negative real permittivity. The most common plasmonic materials are gold and silver. However, many other materials show metal-like optical properties in specific wavelength ranges. The TiO_2_ NPs also exhibit specific properties in response to the light of a particular wavelength [36–38]. Our results are in favor of some previously published scientific studies [12, 13, 15].

The SEM image of the biosynthesized TiO_2_ NPs represented them as irregular and anisotropic (Fig 1B). The particle size histogram shows that the TiO_2_ NPs were between 20–100 nm while most of the nanoparticles were between 40–65 nm (Fig 1B Inset). A previous study reported the spherical shape of the TiO_2_ NPs by using the green synthetic approach while some nanoparticles were irregularly shaped and the size range was between 25–100 nm [12].

The elemental constitution of the TiO_2_ NPs was confirmed by EDX analysis which established the presence of TiO_2_ in the phyto-fabricated nanoparticle sample. The absorption peaks of titanium were reported in the range of 4–5 keV (Fig 1C). The presence of other elements such as carbon in the nanoparticles is because of the presence of various organic phytometabolites in the plant leaf extract used to reduce the TiO_2_ salt.

The XRD analysis revealed the diffractions peaks at 25.2°, 38.2°, 39.0°, 39.8°, 48.3°, 54.5°, 56.3°, 63.3°, 69.6°, 70.9°, 77.0° which are analogous to (101), (103), (004), (112), (200), (105), (211), (116), (220), (605), (215) Miller indices. The JCPDS card number is 04–0783. The presence of the peak pattern confirms the crystalline structure of TiO_2_ NPs. The XRD pattern revealed the crystalline nature of the TiO_2_ NPs and the average size of TiO_2_ NPs was calculated as around 24.58 nm (Fig 1D).

#### 3.1.1 FTIR analysis of TiO_2_ NPs

**Fig 2:**
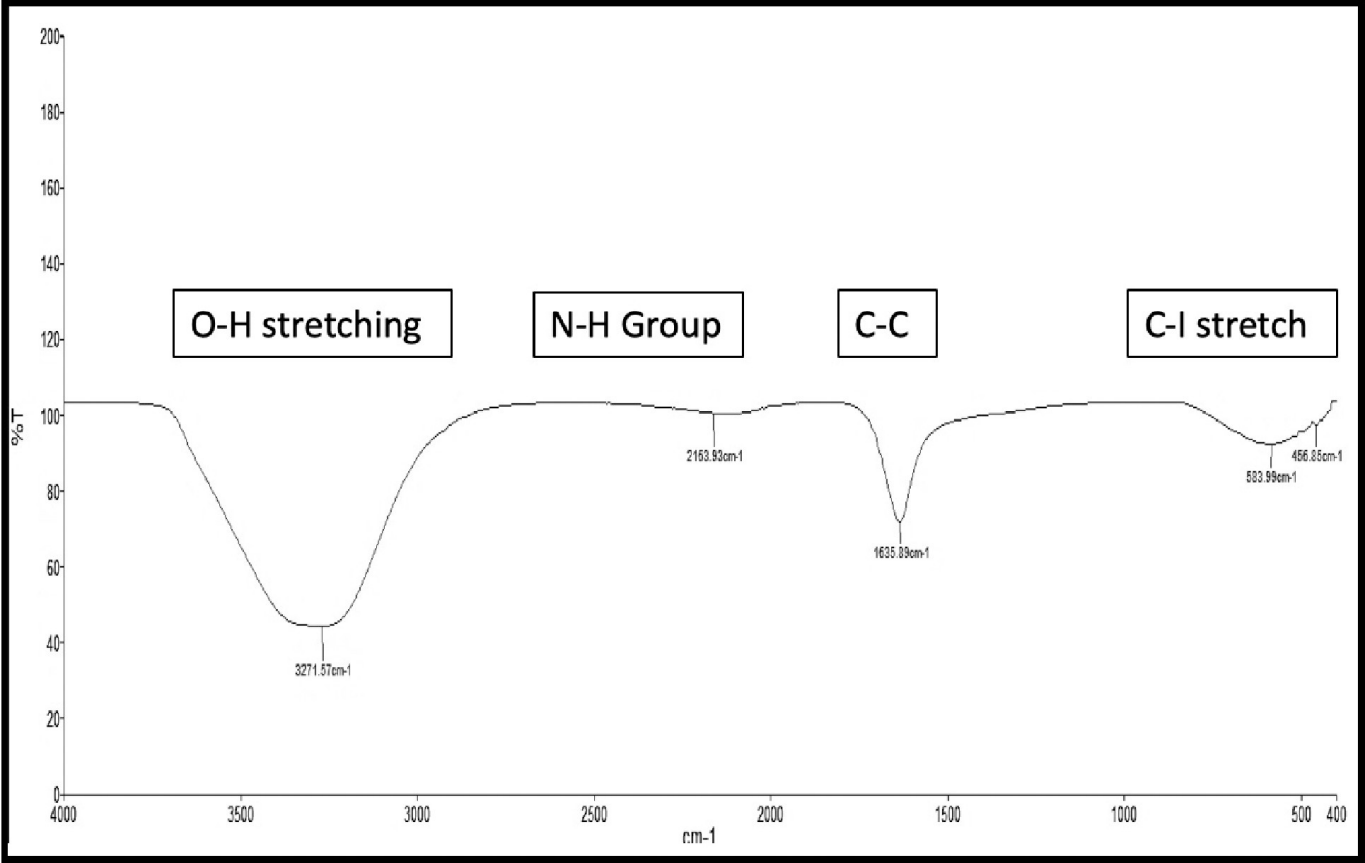
FTIR spectrum of the TiO_2_ NPs.

The FTIR spectroscopy analysis of prepared TiO_2_ NPs showed the existence of different functional groups that were recorded at various wavelengths (Fig 2). The broad absorption peak was observed at 3271.57 cm^-1^ indicates the presence of higher concentrations of alcohol, phenols with O-H stretching. The peak at 2163.93 cm^-1^ represents the presence of ammonium ions with N-H stretches. A medium peak at 1635.89 cm^-1^ represents acyclic compounds with C-C stretches. A low peak at 583.99 cm^-1^ shows aliphatic iodo-compounds with C-I stretch. Similar peaks were reported by the reference [39] for TiO_2_ NPs synthesized by using *Ageratina altissima* (L.) leaf extract which showed peaks at 3377, 2924, 2854, 1621 and 672 cm^-1^ for their representative biomolecules. The present results of the XRD analysis of TiO_2_ NPs synthesized by *Moringa oliefera* leaf extract also matched with the functional groups of *Jatropha curcas* leaf extract reported earlier by the reference [40].

### 3.2 Assessment of the disease severity

**Fig 3:**
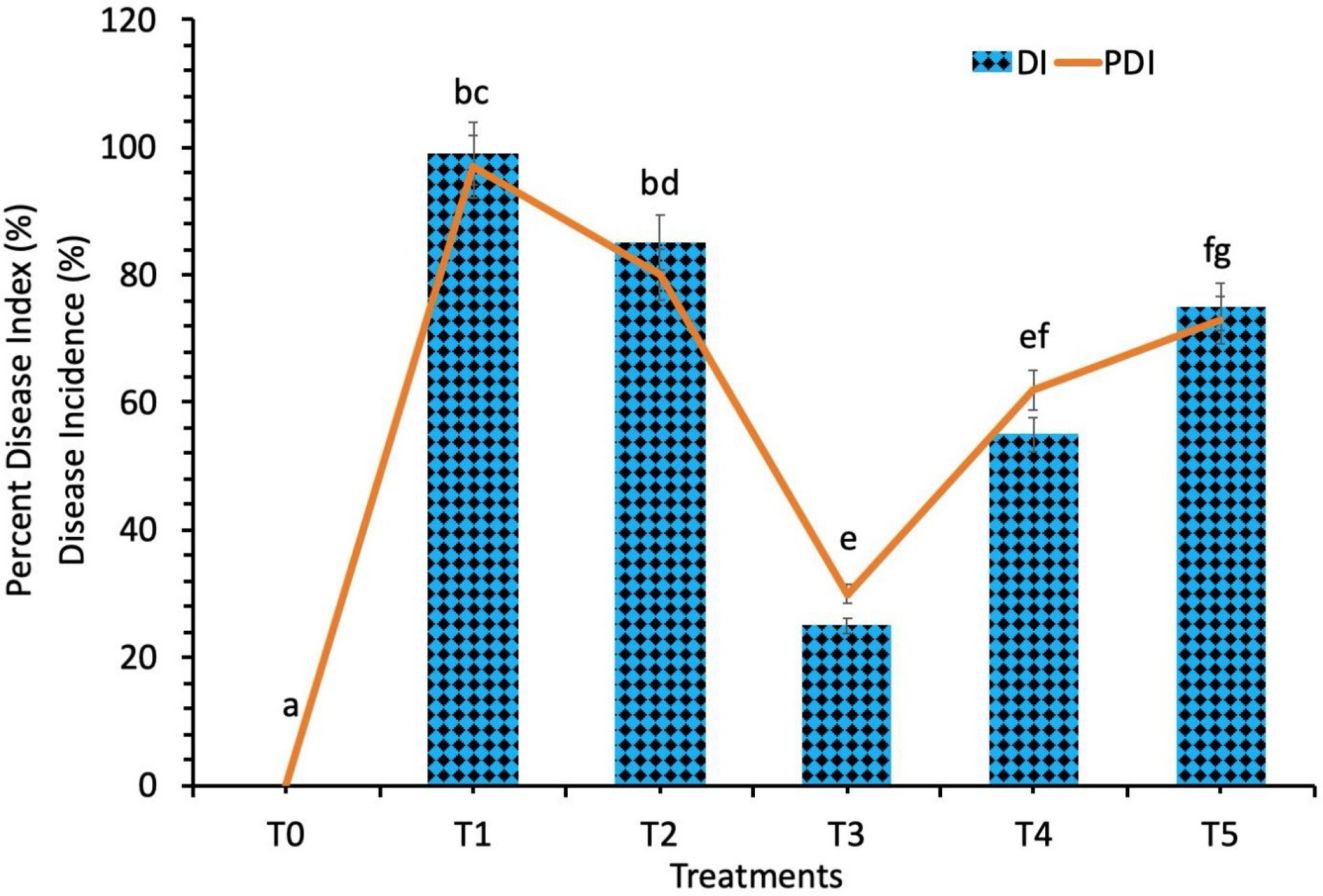
Disease incidence and percent disease index of the wheat plants inoculated with *Bipolaris sorokiniana*. Treatment; T0: *Healthy Plants*, T1: *Plants Infected with Pathogen*, T2: *20 mg/L*, T3: *40 mg/L*, T4: *60 mg/L* and T5: *80 mg/L*. Note: Different alphabets represent that the results are significantly different (*p < 0*.*05*).

The disease incidence and the percent disease index was recorded against the spot blotch disease (*Bipolaris sorokiniana)* by using the different concentrations of biosynthesized TiO_2_ NPs at different day intervals (day 5, day 10, day 15, day 20, day 25 and day 30). The effect of the biosynthesized TiO_2_ NPs on the occurrence of the spot botch disease showed great variance depending upon the number of days passed after the application of various concentrations of the biosynthesized TiO_2_ NPs. It was observed that none of the concentrations of biosynthesized TiO_2_ NPs completely inhibited the spot blotch infection; however, the severity of the disease was affected by the concentration of TiO_2_ NPs. The disease incidence of the spot blotch disease showed a progressive decrease with time in response to all treatments of biosynthesized TiO_2_ NPs till the 30^th^ day (Fig 3).

The highest disease incidence and percent disease index was recorded in wheat plants under fungal infection (biotic stress) and without TiO_2_ NPs treatment. The lowest value of disease incidence and percent disease index was recorded in the wheat plants under fungal pathogen stress which were treated with a 40 mg/L concentration of the biosynthesized TiO_2_ NPs. The percent disease index values were significantly reduced by 80% in spot blotch infected plants when applied with a 40 mg/L concentration of biosynthesized TiO_2_ NPs (Fig 3). The disease incidence was also reduced at all concentrations of TiO_2_ NPs applied plants while the most significant results were obtained at 40 mg/L concentration of TiO_2_ NPs (Fig 3). It was also revealed that 40 mg/L concentration of TiO_2_ NPs caused a reduction in the disease incidence and percent disease index against spot blotch disease at various time intervals as compared to other concentrations of TiO_2_ NPs. The TiO_2_ NPs have been reported to show significant antimicrobial and antibacterial activity [41] which is of high importance because of the elevated resistance of microbes against antibiotics and also the formation of new resistance genomes [22].

The small size and other morpho-physical attributes of TiO_2_ NPs play a significant role to effectively control and suppress various plant infections mainly caused by pathogens [42]. The antimicrobial activity of the TiO_2_ NPs against *B*. *sorokiniana* is due to their small size. The small size of TiO_2_ NPs helps them to cross the plasma cell membrane. It also results in the destabilization of the fungal outer covering which affects the cell’s homeostasis. The disturbance of the membrane integrity results in the leakage of the cytoplasm and also affect other physiological pathways which leads to the death of the fungal cell. The entry of the TiO_2_ NPs to the fungal nucleus results in the oxidation of the nuclear materials which results in the fragmentation of the DNA and eventually cell death. Other physiological pathways that are responsible for the antimicrobial mechanisms of the TiO_2_ NPs involve, inhibition of enzyme activity, interruption of the activity of ATP synthase, oxidation of mitochondrial enzymes, disruption of electron transport chain, inhibition of cellular signaling pathways and blockage of receptor sites [43].

### 3.3 Assessment of the Plant Morphological Attributes Against *B*. *sorokiniana* Stress in Response to TiO_2_ NPs

A glasshouse experiment was designed to investigate the antifungal effects of the TiO_2_ NPs against the biotic stress of the spot blotch disease. Various concentrations of TiO_2_ NPs such as 20 mg/L, 40 mg/L, 60 mg/L and 80 mg/L were applied thrice on the plants i.e. once before inoculation, then 5 days after inoculation and after an interval of 10 days. Plants were subjected to analysis for the *in situ* morphological and physicochemical attributes assessment.

**Fig 4:**
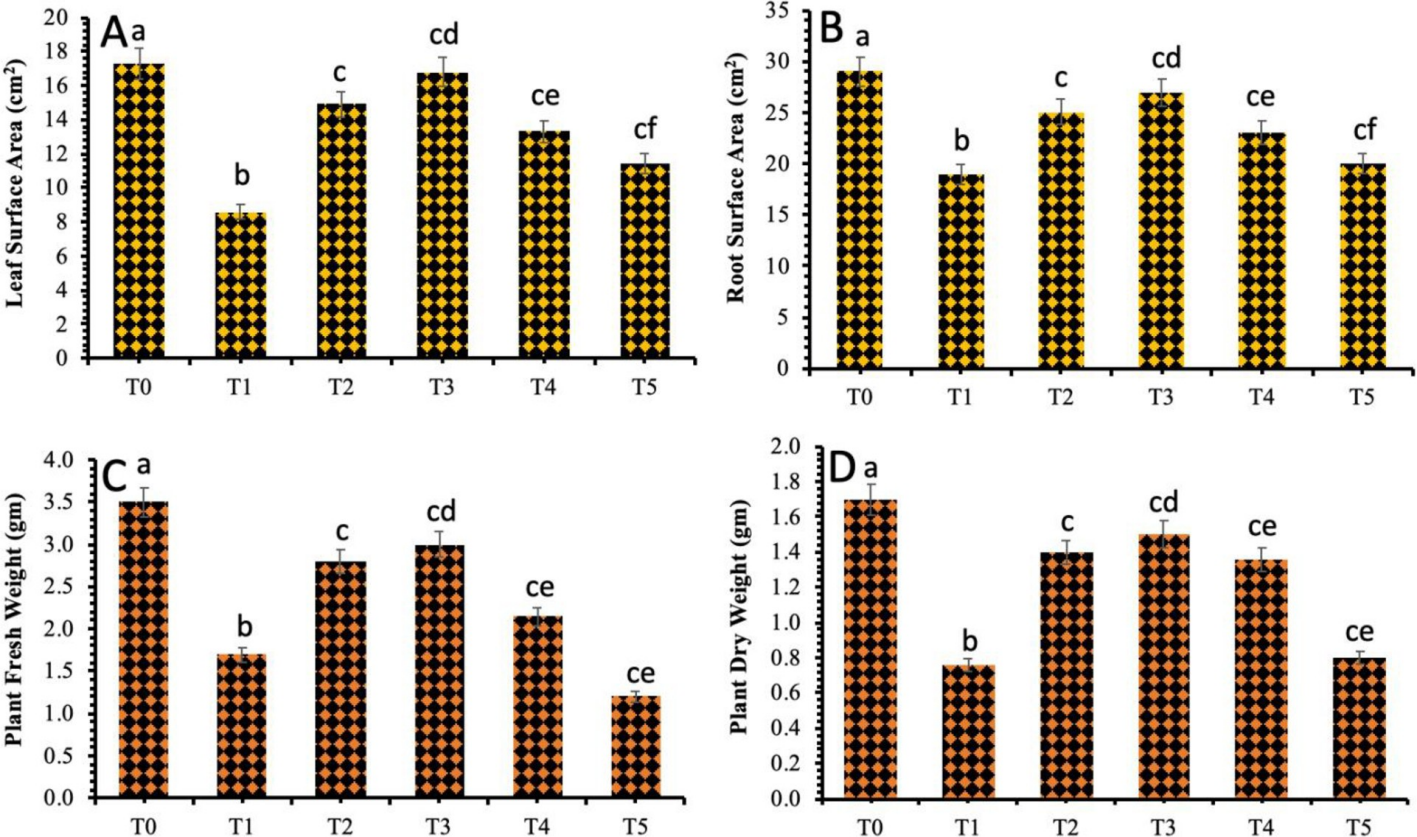
Effect of different treatments of TiO_2_ NPs on (a) Leaf surface area (b) Root surface area (c) Plant fresh weight and (d) Plant dry weight. Treatment; T0: *Healthy Plants*, T1: *Plants Infected with Pathogen*, T2: *20 mg/L*, T3: *40 mg/L*, T4: *60 mg/L* and T5: *80 mg/L*. Note: Different alphabets represent that the results are significantly different (*p < 0*.*05*).

A noticeable decrease in the plant agro-morphological attributes was observed in wheat plants inoculated with the *Bipolaris sorokiniana*. The effects of various treatments of the biosynthesized TiO_2_ NPs on leaf and root surface area varied greatly, mainly due to the application of different concentrations of the synthesized TiO_2_ NPs and the time in terms of days passed after inoculation (Fig 4). The data revealed that 40 mg/L concentration of TiO_2_ NPs had a positive impact on the leaf and root surface area as compared to the other treatments of the TiO_2_ NPs. The maximum leaf surface area (16.6 cm^2^) and root surface area (28 cm^2^) was observed when the spot blotch infected wheat plants were exogenously applied with a 40 mg/L concentration of biosynthesized TiO_2_ NPs. The 60 mg/L and 80 mg/L concentrations of TiO_2_ NPs were revealed to reduce the leaf surface area and root surface area (Fig 4A and 4B).

The plant fresh and dry weight was also significantly decreased in the plants infected with spot blotch disease (Fig 4C and 4D). However, the application of TiO_2_ NPs improved both plant fresh weight and dry weight. The minimum value of the plants’ fresh weight and dry weight was recorded at 1.76 gm and 0.87 gm respectively in the fungal infected plants. However, the significant increase in the plant fresh and dry weight was documented at 3.35 gm and 1.9 gm respectively in plants that were treated with exogenously applied 40 mg/L of TiO_2_ NPs. The results also revealed a further decrease in the plant fresh and dry weight at 80 mg/L of TiO_2_ NPs which suggested the toxic effect of TiO_2_ NPs at higher concentrations (Fig 4C and 4D).

The current research data showed a considerable increase in the fresh weight and the dry weight of the wheat plants under biotic stress in response to 40 mg/L concentration of TiO_2_ NPs. Whereas, the values decreased as the concentration was increased to 60 mg/L and 80 mg/L. The toxic impacts of NPs on higher concentrations vary with the size of nanoparticles, concentration and time interval application of nanoparticles on plants [44]. The findings of the ongoing study are in accordance with the reference [45], who reported that the lower concentrations of TiO_2_ NPs considerably elevated the fresh weight of wheat shoots whereas the higher concentration of TiO_2_ NPs had imparted the growth and development of the plants. An improvement in the growth characters was also reported when TiO_2_ NPs were applied exogenously on *Calendula officinalis* [46].

### 3.4 Assessment of the plant yield attributes against *B*. *sorokiniana* stress in response to TiO_2_ NPs

**Fig 5:**
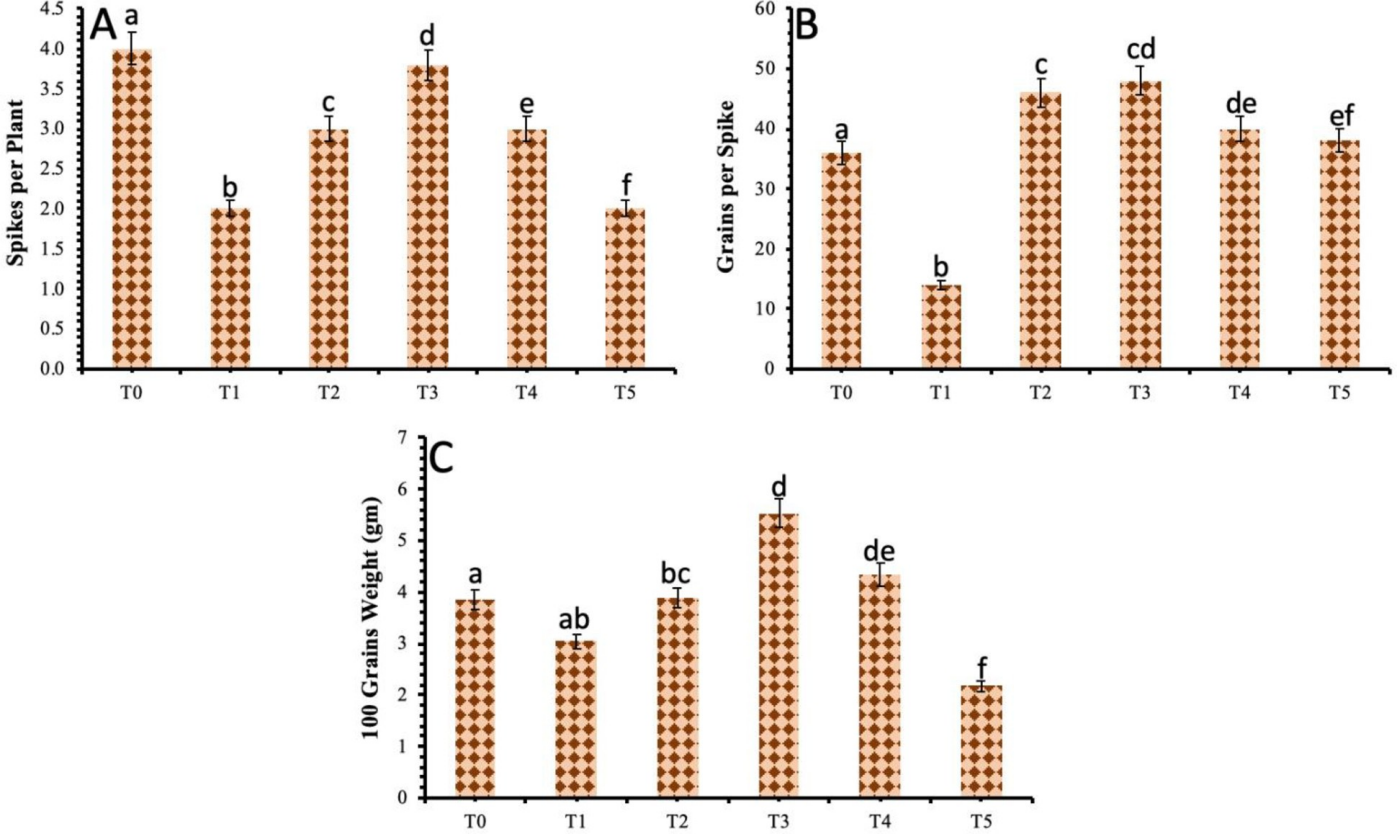
Effect of different treatments of TiO_2_ NPs on **(a)** Spikes per plant **(b)** Grains per spike and **(c)** 100 grains weight. Treatment; T0: *Healthy Plants*, T1: *Plants Infected with Pathogen*, T2: *20 mg/L*, T3: *40 mg/L*, T4: *60 mg/L* and T5: *80 mg/L*. Note: Different alphabets represent that the results are significantly different (*p < 0*.*05*).

The results revealed a decrease among yield attributes of the wheat plants under *Bipolaris sorokiniana* stress. There was a considerable decrease in spikes per plant, grains per spike and the weight of 100 grains in comparison to the control plant. The results also showed an improvement in the overall production of the wheat plant with the exogenous application of biosynthesized TiO_2_ NPs on infected plants. However, the most significant improvement in grains in each spike, the weight of 100 grains and the spike in each plant were shown in plants given 40 mg/L concentration of TiO_2_ NPs under biotic stress (Fig 5A–5C).

The biotic stress greatly affects the physiological and biochemical pathways of plants which in turn affects the plants’ morphology and severely affects the yield of the crop. In this study, the wheat plants under biotic stress of fungal diseases showed a remarkable decrease in the number of grains in each spike, 100 seeds weight in grams and number of spikes in each plant. However, the TiO_2_ NPs application showed an improvement in all attributes of plants which in turn imparted an improvement in yield parameters. The maximum positive results were observed in wheat plants under biotic stress when they were sprayed with a 40 mg/L concentration of TiO_2_ NPs. The higher concentrations (60 mg/L and 80 mg/L) did not show any improvement in yield attributes. The reference [47] reported that TiO_2_ has the capability to regulate the activity of enzymes associated with nitrogen metabolism, hence facilitate plants to obtain more nutrients. Also, the TiO_2_ NPs convert the nitrogen to organic nitrogen in the form of proteins and chlorophyll pigments which ultimately increase the biomass and dry weight of the plants. The reference [46] reported an increase in crop yield when TiO_2_ NPs were sprayed at the reproductive parts of the plants. The foliar application of TiO_2_ NPs on wheat plants also showed an increase in starch and gluten contents due to increased rubisco activity promoted by TiO_2_ NPs, thus enhanced photosynthetic activity which in turn increases the yield [48]. The reference [49] also reported an increase in soybean yield because of increased absorption of water by the plants after treating with TiO_2_ NPs.

### 3.5 Assessment of plant physicochemical parameters against *B*. *sorokiniana* stress in response to TiO_2_ NPs

**Fig 6:**
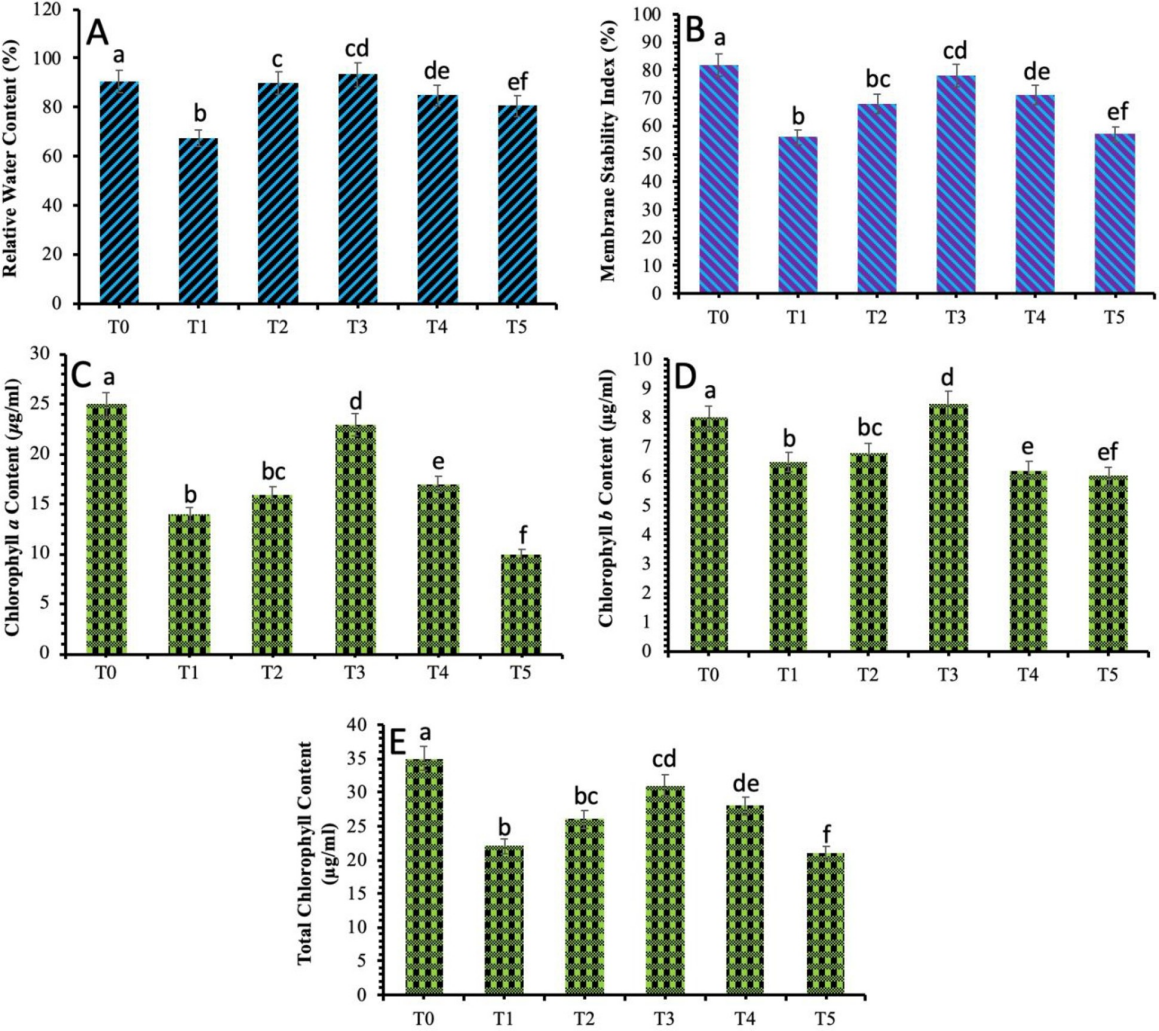
Effect of different treatments of TiO_2_ NPs on the physiological parameters of wheat plants under spot blotch stress (a) Relative water content (b) Membrane stability index (c) Chlorophyll *a* content (d) Chlorophyll *b* content (e) Total chlorophyll content. Treatment; T0: *Healthy Plants*, T1: *Plants Infected with Pathogen*, T2: *20 mg/L*, T3: *40 mg/L*, T4: *60 mg/L* and T5: *80 mg/L*. Note: Different alphabets represent that the results are significantly different (*p < 0*.*05*).

The physicochemical attributes of the wheat plants were analyzed to investigate the antifungal effects of the biosynthesized TiO_2_ NPs against spot blotch disease in wheat. The data recorded showed that the relative water content of the wheat plants under biotic stress was noticeably less in comparison to the control plants. The application of TiO_2_ NPs produced eloquent results and an increase in the relative water content in fungal stressed plants was observed. The highest reading was observed in the plants which were applied with the 40 mg/L of biosynthesized TiO_2_ NPs and showed a decrease in the relative water content percentage as the concentration of the TiO_2_ NPs was tend to increase from 60 mg/L to 80 mg/L (Fig 6A).

The decrease in membrane stability index was recorded in the plants inoculated with spot blotch disease as compared to the control plant. The data regarding foliar applications of biosynthesized TiO_2_ NPs showed that 40 mg/L concentration of TiO_2_ NPs improved the membrane stability up to 29% in infected plants (Fig 6B).

The biotic stress of the spot blotch in wheat plants also resulted in a significant decrease in the chlorophyll *a*, chlorophyll *b* and total chlorophyll content. However, the plants exposed to 40 mg/L of biosynthesized TiO_2_ NPs showed an improvement in the chlorophyll *a*, chlorophyll *b* and total chlorophyll content. The total chlorophyll content was increased from 25 μg/mL in stressed plants to 31 μg/mL in plants that were treated with 40 mg/L of biosynthesized TiO_2_ NPs. (Fig 6C–6E).

A positive impact of the TiO_2_ NPs on the main pigments related to photosynthesis in maize was also described earlier [46]. A previous research report expressed enhanced growth attributes, dry weight values, pigments related to photosynthesis and increased rate of photosynthesis [50]. The authors suggested that the increased chlorophyll content imparted a positive impact on the photosynthesis rate and lead to the increased production of carbohydrates and ultimately increase the fresh weight and dry weights [51].

TiO_2_ NPs have been reported to produce more carbohydrates, thus promotes growth and photosynthesis rate in plants [52]. They have also shown photocatalytic activity to degrade different types of pesticides [42]. The photocatalytic property of TiO_2_ NPs can be of significance in the protection of plants against pathogens as it does not produce toxic and harmful compounds hence renders high pathogen disinfection ability [42, 53].

### 3.6 Assessment of the non-enzymatic attributes of wheat plants against *B*. *sorokiniana* stress in response to TiO_2_ NPs

**Fig 7:**
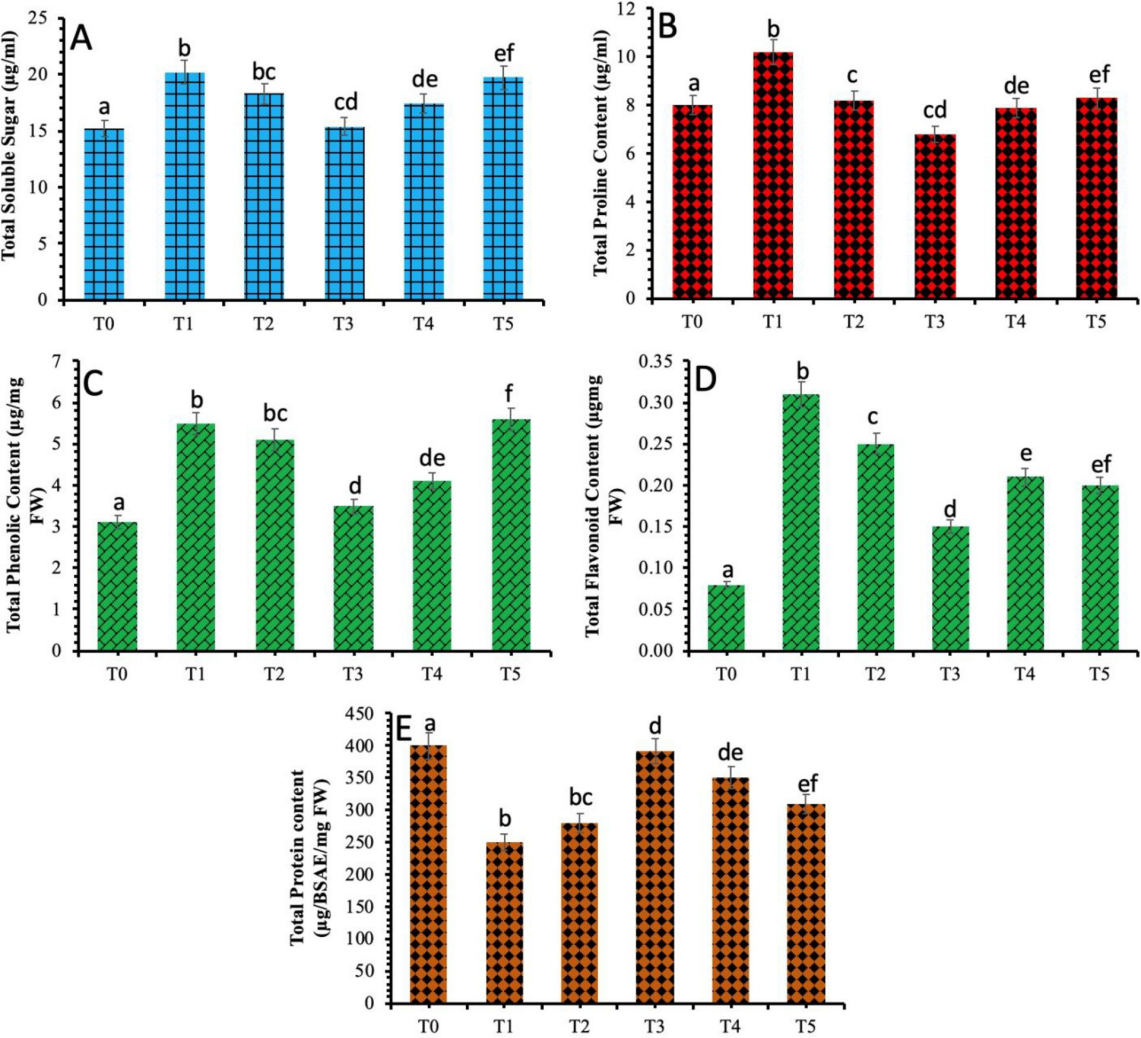
Effect of different treatments of TiO_2_ NPs on non-enzymatic parameters of wheat plants under spot blotch stress (a) Total soluble sugar (b) Total proline content (c) Total phenol content (d) Total flavonoid content (e) Total protein content. Treatment; T0: *Healthy Plants*, T1: *Plants Infected with Pathogen*, T2: *20 mg/L*, T3: *40 mg/L*, T4: *60 mg/L* and T5: *80 mg/L*. Note: Different alphabets represent that the results are significantly different (*p < 0*.*05*).

According to the results, a considerable amount of the soluble sugars were accumulated in the plants under the biotic stress of spot blotch (20.2 μg/mL) in comparison to the control plants. However, exogenous spraying of TiO_2_ NPs had shown a significant decline in the quantity of soluble sugar in the infected plants. The most evident decrease in the content of soluble sugar was recorded in the wheat plants treated with 40 mg/L of TiO_2_ NPs (Fig 7A). A considerable increase in proline concentration was observed in wheat plants under spot blotch stress. However, the application of TiO_2_ NPs proved to be effective and improve the proline content of the infected plants. Proline content was significant (10.2 μg/mL) in infected plants and decreased significantly in the plants treated with 40 mg/L of TiO_2_ NPs (Fig 7B).

The phenolic and flavonoid content was also investigated in the plants under spot blotch stress in response to the biosynthesized TiO_2_ NPs (Fig 7C and 7D). It was observed that the total content of phenolic compounds was increased in wheat plants provided with biotic stress (5.5 μg/mg FW) and decreased considerably in wheat plants applied with biosynthesized TiO_2_ NPs. A maximum decrease was observed in the plants applied with 40 mg/L of TiO_2_ NPs. The total content of flavonoids also showed an increase in plants under biotic stress of spot blotch and decreased considerably in plants provided with TiO_2_ NPs. The most significant decrease in the flavonoid amount was observed in the plants provided with 40 mg/L of TiO_2_ NPs. The total flavonoid content showed dependency and linear correlation with the total phenolic content. It was observed previously that the TiO_2_ NPs triggered the activities of catalase and glutathione reductase in the water thyme plant [19]. A considerable difference in amide, carbohydrates and lignin values were observed in cucumber plants when treated with TiO_2_ NPs which suggested that TiO_2_ NPs exposure can bring the alterations at the macromolecular level in cucumber fruits [52].

The results also revealed a decrease in the content of total proteins in the plants infected with spot blotch. However, the content of total protein showed an evident increase after the TiO_2_ NPs application. The most significant increase in total protein content was documented in wheat plants provided by 40 mg/L of TiO_2_ NPs (Fig 7E).

## 4. Conclusion

Herein, we reported a one-pot and eco-friendly method for the biosynthesis of titanium dioxide nanoparticles using the reducing and stabilizing potential of *Moringa oleifera* leaf aqueous extract. The biogenic titanium dioxide nanoparticles were reported to be irregular in shape and existed in between 20 to 100 nm stabilized by O-H, N-H, C-C and C-I functional groups. The measurement of the disease incidence and percentage disease index reported that the severity of the disease decreased in response to the foliar applications of titanium dioxide nanoparticles over time. The biosynthesized titanium dioxide nanoparticles showed the ability to develop resistance in the wheat plants against *Bipolaris sorokiniana*, which is responsible to cause spot blotch disease. The morphological parameters were reported in terms of plant fresh and dry weight, leaf and root surface area and yield parameters while physiological parameters were recorded as relative water content, membrane stability index, chlorophyll contents, soluble sugar, protein, proline, flavonoid and phenolic contents and they were, stabilized to induce disease tolerance in wheat plants in response to the 40 mg/L foliar applications of biogenic titanium dioxide nanoparticles. The findings of the current study establish a foundation for the in-depth analysis of the effectiveness and toxicity of the nanoparticles and alterations at the molecular level in response to the biotic stress.
